# Modeling the Novel Coronavirus (SARS-CoV-2) Outbreak in Sicily, Italy

**DOI:** 10.3390/ijerph17144964

**Published:** 2020-07-09

**Authors:** Andrea Maugeri, Martina Barchitta, Sebastiano Battiato, Antonella Agodi

**Affiliations:** 1 Department of Medical and Surgical Sciences and Advanced Technologies “GF Ingrassia”, University of Catania, 95123 Catania, Italy; andrea.maugeri@unict.it (A.M.); martina.barchitta@unict.it (M.B.); 2 Department of Mathematics and Computer Science, University of Catania, 95123 Catania, Italy; battiato@dmi.unict.it; 3 Azienda Ospedaliero-Universitaria “Policlinico-Vittorio Emanuele”, 95123 Catania, Italy

**Keywords:** novel coronavirus, COVID-19, epidemic model, epidemiology

## Abstract

Italy was the first country in Europe which imposed control measures of travel restrictions, quarantine and contact precautions to tackle the epidemic spread of the novel coronavirus (SARS-CoV-2) in all its regions. While such efforts are still ongoing, uncertainties regarding SARS-CoV-2 transmissibility and ascertainment of cases make it difficult to evaluate the effectiveness of restrictions. Here, we employed a Susceptible-Exposed-Infectious-Recovered-Dead (SEIRD) model to assess SARS-CoV-2 transmission dynamics, working on the number of reported patients in intensive care unit (ICU) and deaths in Sicily (Italy), from 24 February to 13 April. Overall, we obtained a good fit between estimated and reported data, with a fraction of unreported SARS-CoV-2 cases (18.4%; 95%CI = 0–34.0%) before 10 March lockdown. Interestingly, we estimated that transmission rate in the community was reduced by 32% (95%CI = 23–42%) after the first set of restrictions, and by 80% (95%CI = 70–89%) after those adopted on 23 March. Thus, our estimates delineated the characteristics of SARS-CoV2 epidemic before restrictions taking into account unreported data. Moreover, our findings suggested that transmission rates were reduced after the adoption of control measures. However, we cannot evaluate whether part of this reduction might be attributable to other unmeasured factors, and hence further research and more accurate data are needed to understand the extent to which restrictions contributed to the epidemic control.

## 1. Introduction

The novel coronavirus (SARS-CoV-2) that emerged in Wuhan (China) at the end of 2019 quickly spread to another 213 countries, as of 13 April 2020. On the same date, there were 159,516 confirmed SARS-CoV-2 cases with 20,465 deaths in Italy [1]. It is worth mentioning that Italy was the first country in Europe to put its entire population on lockdown [2]. On 10 March, the Italian Government imposed control measures to the entire country, including travel restrictions, quarantine and contact precautions [3,4]. On 23 March, further restrictive measures, which included the shutdown of all non-strategic production in the whole Italian territory, have been adopted [3,4]. Such efforts to contain the virus are still ongoing in all the Italian regions, however, uncertainties regarding SARS-CoV-2 transmissibility and virulence make it difficult to evaluate the effectiveness of restrictions. A major source of uncertainty regards the proportion of unreported cases, a critical epidemiological characteristic that might bias the interpretation of the epidemic curve. Indeed, if one looks at only confirmed data, there has been a reduction in the epidemic spread in Italy after restrictions. Yet, mounting evidence suggested that undocumented infections could greatly contribute to SARS-CoV-2 spread [5,6,7]. Moreover, several outbreaks occurred at different times scattered throughout the Italian territory [1]. For this reason, it is necessary to evaluate the epidemic curve on a region-by-region basis, using data that are less prone to ascertainment bias [8], such as such as those related to hospital admission and deaths [7].

In this study, we employed a Susceptible-Exposed-Infectious-Recovered-Dead (SEIRD) model to assess SARS-CoV-2 transmission dynamics in Sicily (South Italy). The first aim was to estimate the unknown parameters of the model before restrictions of 10 March, also assessing the possibility of unreported cases. We further modeled the epidemic to evaluate changes in transmission rates after the adoption of control measures on 10 and 23 March 2020.

## 2. Materials and Methods

### 2.1. Formulating the SEIRD Model

**Figure 1:**
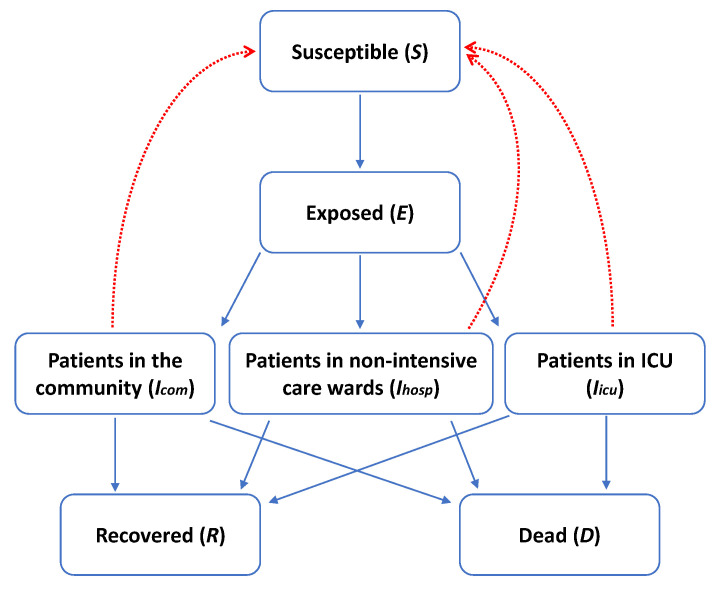
The employed Susceptible-Exposed-Infectious-Recovered-Dead (SEIRD) epidemic model for novel coronavirus (SARS-CoV-2) epidemic in Sicily, Italy. This diagram summarizes the flow of individuals across SEIRD compartments (blue lines); red dotted lines, instead, represent the potential transmission of SARS-CoV-2 from infectious to susceptible individuals.

As described in a previous study [7], we modeled the epidemic curve using a Susceptible-Exposed-Infectious-Recovered-Dead (SEIRD) model, where *S*(*t*), *E*(*t*), *I*(*t*), *R*(*t*), and *D*(*t*) were the number of susceptible (*S*), exposed (*E*) (i.e., infected but not yet be infectious), infectious (*I*), recovered (*R*) and dead (*D*) individuals at each time (*t*). However, to take into account different transmission dynamics in the community and in hospitals, we separated the infectious state into three classes: patients with very mild or no symptoms in the community (*I_com_*), those with mild clinical presentation admitted to non-intensive care wards (*I_hos_*), and those with severe disease who required Intensive Care Unit (ICU) hospitalization (*I_icu_*) (Figure 1).

Accordingly, the model was defined by the following system of ordinary differential equations:

(1) $$\frac{dS\left(t\right)}{dt}=-\frac{\left(\beta {}_{com}I{}_{com}\left(t\right)+\beta {}_{hos}I{}_{hos}\left(t\right)+\beta {}_{icu}I{}_{icu}\left(t\right)\right)\;S}{N}$$

(2) $$\frac{dE\left(t\right)}{dt}=\frac{\left(\beta {}_{com}I{}_{com}\left(t\right)+\beta {}_{hos}\;I{}_{hos}\left(t\right)+\beta {}_{icu}\;I{}_{icu}\left(t\right)\right)\;S}{N}-\sigma E\left(t\right)$$

(3) $$\frac{dI\left(t\right)}{dt}=\sigma E\left(t\right)\;\upsilon {}_{com}+\sigma E\left(t\right)\;\upsilon {}_{hos}+\sigma E\left(t\right)\;\upsilon {}_{icu}-\gamma I\left(t\right)$$

(4) $$\frac{dR\left(t\right)}{dt}=\gamma \left(1-\mu {}_{com}\right)\;I{}_{com}\left(t\right)+\gamma \left(1-\mu {}_{hos}\right)\;I{}_{hos}\left(t\right)+\gamma \left(1-\mu {}_{icu}\right)\;I{}_{icu}\left(t\right)$$

(5) $$\frac{dD\left(t\right)}{dt}=\gamma \mu {}_{com}I{}_{com}\left(t\right)+\gamma \mu {}_{hos}I{}_{hos}\left(t\right)+\gamma \mu {}_{icu}I{}_{icu}\left(t\right)$$

where:

- *N* was the total population given by the sum of each state;
- *β_com_*, *β_hos_*, and *β_icu_* were the transmission rates in the three infectious categories;
- *σ* was the infection rate (i.e., the inverse of the incubation period) assumed to be the same for each infectious category;
- *υ_com_*, *υ_hos_*, and *υ_icu_* were the probabilities that each exposed individual progressed to *I_com_*, *I_hos_* or *I_icu_*;
- *γ* was the removing rate (i.e., assumed to be the inverse of the infectious period between onset of symptoms and recovering/death);
- *μ_com_*, *μ_hos_*, and *μ_icu_* were the probabilities of dying among infectious individuals.

In brief, Equations (1) and (2) regulated the flow of patients from *S* to *E* compartment according to the number of *S* and *I* individuals at each time (*t*), the transmission rates *β_com_*, *β_hos_*, and *β_icu_*, and the total population *N*. Specifically, *S* individuals could become *E* after contact with *I* individuals. Equation (3) regulated the flow of patients from *E* to *I* compartment according to the number of *E* individuals at each time (*t*), the infection rate *σ*, and the probabilities that each *E* individual progressed to *I_com_*, *I_hos_* or *I_icu_*. Thus, each *E* individual could progress to *I_com_*, *I_hos_* or *I_icu_* with different probabilities. Equations (4) and (5) regulated the flow of patients from *I* to *R* or *D* compartments according to the number of *I* individuals at each time (*t*), the removing rate and the probabilities of dying or surviving among *I* individuals. Notably, at each time (*t*), the sum of Equations (4) and (5) was equal to *γI*.

### 2.2. Fitting the Model to the Reported Number of ICU Patients and Deaths

The SEIRD model was formulated to estimate the unknown parameters and to reflect the epidemic curve in a way that was consistent with reported data. However, as discussed in our previous work [7], stringent testing strategies that have been adopted in Italy and other countries might have caused an underestimation of infectious individuals, especially those with mild or no symptoms. To address the uncertainty on reported SARS-CoV-2 infections, we fitted our model to reported data on the daily number of patients hospitalized in ICU and on the number of deaths, which were certainly less prone to be affected by ascertainment bias [8]. These data were obtained from Italy’s Civil Protection department of the Italian Ministry of Health from 24 February to 13 April 2020 [1], and reported in Table S1. In particular, we collected all the data but we fitted our model to minimize the following objective function (6):

(6) $$F=\sum_{t=1}^{n{}_{icu}}\;\left(I{}_{icu-obs}-I{}_{icu-est}\right)\left(t\right)+\sum_{t=1}^{n_{deaths}}\;(D_{obs}-D_{est}){\left(t\right)}_{\;}$$

where:

- *I_icu−obs_* and *I_icu−est_* were the number of observed and estimated ICU patients at each time (*t*) from 24 February to 17 March. Thus, in the baseline scenario, *n_icu_* was set to 23 (i.e., the number of days between start and end dates of the model fitting on ICU patients);
- *D_obs_* and *D_est_* were the number of observed and estimated deaths at each time (*t*) from 24 February to 24 March. Thus, in the baseline scenario, *n_deaths_* was set to 30 (i.e., the number of days between start and end dates of the model fitting on deaths).

In fact, given the lag of 1–2 weeks between the adoption of restrictions (i.e., first restrictions were adopted on 10 March) and their impact on infection and death trends, we were confident in using the above-mentioned periods to fit our model. A summary of this process and data used to fit the model are reported in Figure S1 and Table S1, respectively. To estimate the unknown parameters, we employed a Sequential Quadratic Programming (SQP) algorithm for large-scale constrained optimization, which solved a sequence of optimization subproblems to minimize the objective function (6) satisfying all the constraints [9]. This algorithm has already been used for modeling purposes in the context of SARS-CoV-2 epidemic [10]. The model is based on 10 predefined parameters (i.e., *N*, *S*, *E*, *I_com_*, *I_hos_*, *I_icu_*, *R*, *D*, *σ*, and *γ*) and 9 parameters to be estimated (i.e., *β_com_*, *β_hos_*, *β_icu_*, *υ_com_*, *υ_hos_*, *υ_icu_*, *μ_com_*, *μ_hos_*, and *μ_icu_*). The modeling started 5 days before the first case was announced in Sicily on 25 February, with one infectious individual in the community. This lag time corresponded to the incubation period of SARS-CoV-2 estimated by Li et al. [11]. The entire set of assumptions and constraints was defined based on the current knowledge on SARS-CoV-2 features and looking at the epidemic curve in Sicily (Table 1). Among these, the infection rate was assumed to be 1/5.2 days according to Li et al. [11]; the removing rate was set to 1/12 days based on infectious and hospitalization periods reported by previous studies in China and Italy [6,12,13]. However, there was no consensus on the removing rate, and hence its uncertainties were further considered in the sensitivity analysis. The above process was iterated 1000 times on randomly generated samples from the distribution functions of reported ICU patients and deaths to obtain 95% Confidence Intervals (CIs). Specifically, the reported number of ICU patients and deaths followed two different third-degree polynomials with R^2^ = 0.987 and R^2^ = 0.984, respectively.

### 2.3. Modeling the SARS-CoV-2 Transmission after the Adoption of Control Measures

We further modeled the transmission of SARS-CoV-2 in Sicily after 10 March, when the control measures of travel restrictions, self-quarantine and contact precautions have been advocated by the Italian Government. To do that, we re-estimated the transmission rates in the three infectious categories (*β_com_*, *β_hos_*, and *β_icu_*) maintaining the other parameters obtained previously as unchanged. In this case, we fitted the model to the reported number of daily ICU patients from 18 to 27 March and cumulative deaths from 25 March to 2 April. We used a similar approach to take into account more stringent restrictions adopted by the Italian Government on 23 March, by fitting the model to reported daily ICU patients from 28 March to 13 April and cumulative deaths from 3 to 13 April. Again, the reported number of ICU patients and deaths followed different third-degree polynomials with R^2^ ranging from 0.981 to 0.992.

### 2.4. Sensitivity Analysis

Certain parameters of our model could be affected by disease severity, and hence some considerations should be addressed. In the scenario described above, we have already taken into account differences in *β*, *υ*, and *μ* across SARS-CoV-2 patient’s categories. In contrast, *σ* (i.e., the inverse of incubation period) could be assumed as independent of disease severity. For these reasons, we conducted a sensitivity analysis to account for the effect of disease severity on *γ* (i.e., the removing rate). Current uncertainties on this parameter and differences between countries make it difficult to establish a proper value. Our hypothesis was that the infectious period increased with increasing disease severity. Considering the inverse relationship between infectious period and removing rate, we assumed that the removing rate of non-hospitalized patients (*γ_com_*) was higher than that of patients hospitalized in non-intensive care wards (*γ_hos_*). Similarly, the removing rate of patients hospitalized in non-intensive care wards was higher than that of patients hospitalized in ICU (*γ_icu_*)_._ It is worth mentioning that there was still no consensus on the infectious period [12,14,15], and hence its estimates—especially among asymptomatic and mild patients—were not so consistent. In the sensitivity analysis, we assumed three different removing rates for *I_com_*, *I_hos_*, and *I_icu_* according to that used in the baseline scenario. Specifically, we attributed the highest parameter to *I_icu_* (i.e., 1/18 days), an intermediate value for *I_hos_* (i.e., 1/12 days), and the lowest to *I_com_* (i.e., 1/6 days). The highest and the lowest values corresponded to 50% above or below the removing rate used in the baseline scenario. Thus, we performed the analyses by using the above-mentioned removing rates but maintaining the other initial conditions reported in Table 1. Instead, Equations (4) and (5) were replaced with the following:

(7) $$\frac{dR\left(t\right)}{dt}=\gamma {}_{com}\left(1-\mu {}_{com}\right)\;I{}_{com}\left(t\right)+\gamma {}_{hos}\left(1-\mu {}_{hos}\right)\;I{}_{hos}\left(t\right)+\gamma {}_{icu}\left(1-\mu {}_{icu}\right)\;I{}_{icu}\left(t\right)$$

(8) $$\frac{dD\left(t\right)}{dt}=\gamma {}_{com}\mu {}_{com}I{}_{com}\left(t\right)+\gamma {}_{hos}\mu {}_{hos}I{}_{hos}\left(t\right)+\gamma {}_{icu}\mu {}_{icu}I{}_{icu}\left(t\right)$$

## 3. Results

### 3.1. Description of Reported Data

**Figure 2:**
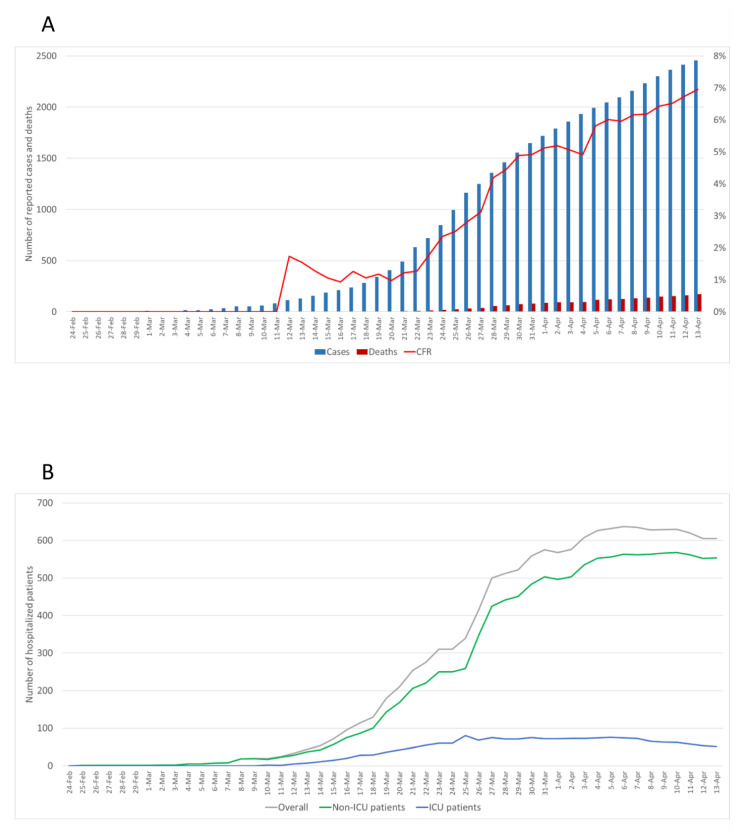
Number of cases, hospitalized patients and deaths in Sicily, Italy, reported by Italy’s Civil Protection from 24 February 2020 to 13 April 2020. (**A**) The bars represent the cumulative number of reported SARS-CoV-2 cases and related deaths while the red line represents the case fatality risk (CFR). (**B**) The grey line represents the daily number of hospitalized patients, while blue and green lines represent the daily number of patients hospitalized in Intensive Care Unit (ICU) or in other wards.

Data on SARS-CoV-2 cases, hospitalized patients and deaths in Sicily, reported by Italy’s Civil Protection from 24 February to 13 April, are shown in Figure 2. As of 13 April 2020, there were 2458 cases, 605 hospitalized patients and 171 deaths. Notably, the daily increment in the number of new cases decreased from 28 March (8.7%) to 13 April (1.7%). Examining the epidemic trend, we also observed that case fatality risk—the number of deaths divided by number of cases—increased from 1.7% to 7.0% as the epidemic spread (Figure 2A). After an exponential growth, instead, the daily number of hospitalized patients reached a steady state from 28 March to 2 April followed by a slight decrement until 13 April. This decline appeared slower for patients hospitalized in non-intensive care wards than those admitted to ICU (Figure 2B).

### 3.2. The Epidemic Curve Prior to Restrictions

**Figure 3:**
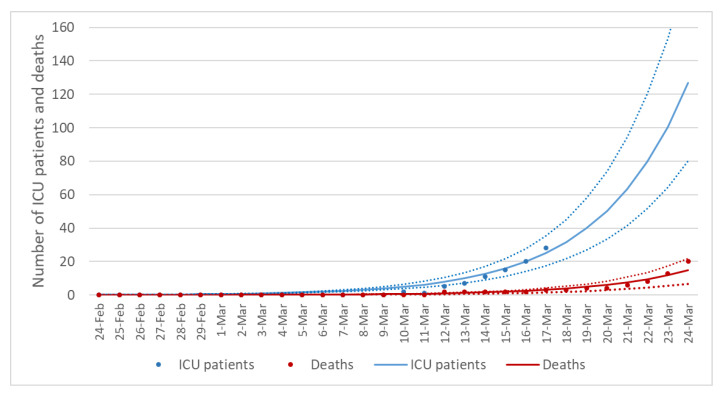
Number of estimated and reported patients in Intensive Care Unit and deaths in Sicily (Italy) from 24 February to 24 March 2020. The SEIRD model was fitted to the reported number of patients admitted to Intensive Care Unit (ICU) and deaths. Blue and red dots represent the number of reported ICU patients and deaths, respectively, while the lines represent their estimates and 95% confidence intervals through the SEIRD model.

Given the uncertainty on data reported by Italy’s Civil Protection, especially those related to confirmed cases, we first fitted the SEIRD model to the reported daily number of ICU patients and cumulative deaths, obtaining an overall good fit between estimated and reported data (Figure 3). The estimated parameters which best explained the reported numbers of ICU patients and deaths are shown in Table 2. Using the best-fitting parameters, we estimated a total of 76 (95%CI = 60–94) SARS-CoV-2 cases on 10 March, which comprised 65 (95%CI = 51–80) infectious individuals, 20 recoveries (95%CI = 17–23), and 1 (95%CI = 0–1) death. With respect to infectious individuals, we estimated 18 (95%CI = 12–24) hospitalized patients, out of which 5 were in ICU (95%CI = 4–6) and 13 were in other wards (95%CI = 10–16). Although these estimates revealed only a small proportion of unreported cases (18.4%; 95%CI = 0–34.0%), the estimated case fatality risk of 0.7% would remain stable as the epidemic spread.

### 3.3. The Effect of Control Measures on Transmission Rates

**Figure 4:**
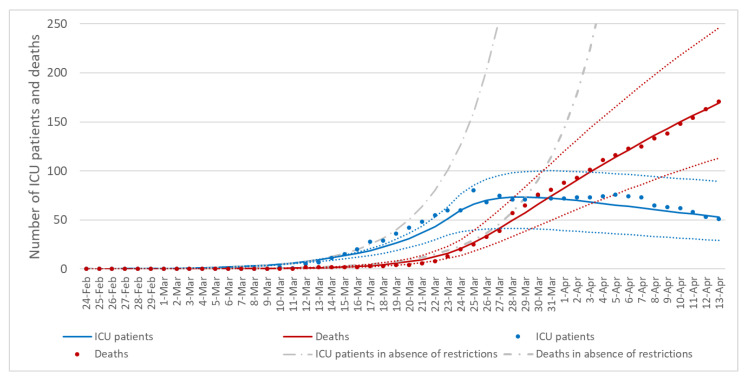
Modeling of control measures adopted on 10 March 2020 which were then intensified on 23 March 2020. Blue and red dots represent the number of reported ICU patients and deaths, respectively, while the lines represent their estimates and 95% confidence intervals through the SEIRD model. Grey lines, instead, represent the estimates in absence of control measures.

We next applied the SEIRD model to evaluate the effect of restrictions adopted by the Italian Government on 10 March, which were then intensified on 23 March (Figure 4). We estimated that transmission rate in the community was reduced by 32% (95%CI = 23–42%) after the first set of restrictions imposed on 10 March 2020. We estimated that also the transmission rates of patients hospitalized in ICU or in other wards were reduced by 10% (95%CI = 5–15%) and 7% (95%CI = 4–10%), respectively. Interestingly, transmission rate in the community was reduced by 80% (95%CI = 70–89%) after control measures adopted on 23 March, while transmission dynamics remained almost unaltered among hospitalized patients (Table 3).

### 3.4. Sensitivity Analysis

Given potential differences in the removing rates across SARS-CoV-2 patient’s categories, we performed a sensitivity analysis and re-evaluated the best-fitting parameters (Table S2). In this scenario, we estimated a total of 74 (95%CI = 58–92) SARS-CoV-2 cases on 10 March, which comprised 3 patients hospitalized in ICU (95%CI = 2–4) and 1 death (95%CI = 0–1). As such, the proportion of unreported cases was 16.2% (95%CI = 0–32.6%). Sensitivity analysis estimated that transmission rate in the community was reduced by 41% (95%CI = 37–46%) after the first set of control measures imposed on 10 March, and by 85% (95%CI = 79–89%) after 23 March 2020.

## 4. Discussion

In this study, we modeled the SARS-CoV-2 epidemic in Sicily (South Italy) to estimate any instances of undocumented infections and to evaluate the impact of control measures adopted by the Italian Government. Our approach stemmed from the hypothesis that looking at reported cases could lead to biased speculations when interpreting data on an epidemic emergency. For this reason, we further adapted our previously described SEIRD model [7] to investigate the SARS-CoV-2 epidemic curve in Sicily, working directly on data on admissions to ICU and deaths. Although uncertainties of available information on SARS-CoV-2 cases do not allow us to validate this model properly, we have already applied a similar approach to Chinese data [7]. A similar hypothesis has been also formulated by a research group from the Imperial College of London, who is investigating the epidemic curve on the basis of deaths observed over time [16]. However, to our knowledge, our study was the first employing also data on admissions to ICU, another event that was certainly less affected by ascertainment bias. Our findings revealed a smaller proportion of unreported cases in Sicily if compared with previous estimates in Italy [17]. A plausible explanation was that the Sicily’s regional health system did not experience the same emergency observed in other Italian regions, such as Lombardy, Emilia Romagna, and Piedmont [18]. However, despite stringent testing strategies, ~20% of cases were not documented on 10 March. This proportion of unreported cases biased the apparent increase in case fatality risk during the epidemic spread. Instead, taking into account the estimated cases, case fatality risk stably stood around 0.7%. In accordance, the probability of dying among ICU patients was consistent with the ICU mortality reported by Grasselli et al. in the Lombardy region [12]. As stated by previous studies [18], indeed, stringent testing strategies adopted in Italy might explain the higher case fatality risk compared to other countries. This raised the need for caution when comparing preliminary estimates between and within countries.

Looking at the data, we noted a gradual flattening of the epidemic curve after the adoption of restrictive measures by the Italian Government in the middle of March. Although it was not clear to what extent the slowdown in the epidemic spread was exclusively attributable to control measures, restrictions have succeeded, at least partially, to contain SARS-CoV-2 transmission. Specifically, measures such as travel restrictions, quarantine and contact precautions might have modified the transmission rate in the community. Indeed, in our study, we estimated that transmission rate in the community decreased by 32% after 10 March and by 80% after 23 March. Beside this, changes in healthcare worker’s behavior such as wearing of facemasks, social distancing, self-isolation when sick contributed by reducing transmission rates in the hospital setting. Indeed, our estimates on the impact of control measures were in line with those reported by Li and colleagues on the Chinese experience [5]. However, it cannot be ruled out that part of this reduction might be attributable to other factors, including improvements in patient management and seasonal variations [19,20]. For these reasons, we decided to not extend our model beyond 13 April 2020. Indeed, the influence of other factors would be increased with increasing the modeling period, while, in our opinion, working on a limited period could partially reduce these effects.

We recognized that our findings were based on an epidemic model with constant parameters and therefore some points should be considered when interpreting our results. Limitations of these kinds of models significantly hinder their long-term predictive application, and hence we exclusively applied our SEIRD model to estimate the proportion of unreported SARS-CoV-2 cases and to evaluate changes in transmission rates after control measures. To do that, we fitted our model to those data that were considered less biased by uncertainty such as admissions to ICU and deaths. Indeed, in a region where ICU bed needs for SARS-CoV-2 had not yet reached saturation, even the reported number of patients in ICU could be considered less biased. However, our model was characterized by other limitations. First, it was based on the assumption that all the Sicilian population was equally susceptible to SARS-CoV-2. Although the number of susceptible individuals could be a fraction of the entire population, a recent study reported a seropositivity of 3–4% in the city of Wuhan, the epicenter of the epidemic [21]. Even considering a similar proportion in Sicily, our estimates would not change, since they depended on the ratio between S individuals and the total population N. Second, the model did not discriminate cases that were imported from other Italian regions or countries and for the longitudinal flow of patients from symptom onset to recovering/death. Third, our estimates were not adjusted for the age structure of the Sicilian population. We recognized that differentiating by age groups could yield to more precise estimates, however, it would be necessary that data to fit the model were stratified by age [22]. Fourth, our model did not allow the evaluation of the independent effect of different control measures adopted (e.g., contact tracing, travel restrictions, quarantine). Moreover, it was also possible that the reduction in the epidemic spread could be partially due to improvements in patient management and seasonal variations. Finally, the model fitting was based on the SQP algorithm, which required a set of predefined constraints for optimization [9], and multiple simulations applied to obtain 95%CIs. Thus, among an infinite set of combinations which could provide an optimal solution, the algorithm identified the best-fitting parameters in a narrow range of possibilities. This partially explains the narrow 95%CIs of our estimates, which might be wider in the absence of predefined constraints. Thus, our approach certainly relied on several assumptions and constraints based on the current knowledge of SARS-CoV-2 characteristics and relevant references published in recent months. However, we cannot rule out some degree of uncertainty of our estimates, which will be further refined with progresses in the knowledge on SARS-CoV-2.

In conclusion, our approach—which assessed the epidemic curve by estimating backwards from the documented admissions to ICU and deaths—appeared to be useful for the investigation of the SARS-CoV2 epidemic. Our model, on the early phase of the outbreak, delineated the characteristics of SARS-CoV2 transmission in a region without major restrictions or control. Further analysis extended after the adoption of control measures, instead, suggested that restrictions might have reduced SARS-CoV2 transmission rates considerably. However, we cannot rule out that part of this reduction might be attributable to other unmeasured factors. Thus, further research and accurate data are needed to understand which control measures contributed to the epidemic control.

## Figures and Tables

**Table 1:** Initial parameters of the SEIRD model.

Initial Model Parameters	Definition	Assumption or Constraints
Starting date ^a^	The first of the modeling	20 February 2020
*N* ^b^	Total population	5,000,000
*S*	Susceptible individuals	4,999,999
*E*	Exposed individuals	0
*I_com_*	Non-hospitalized patients	1
*I_hos_*	Patients hospitalized in non-intensive care wards	0
*I_icu_*	Patients hospitalized in ICU	0
*R*	Recovered	0
*D*	Deaths	0
*β_com_*	Transmission rates in the three infectious categories	0.1 ≤ *β_com_* ≤ 2
*β_hos_*		0.1 ≤ *β_hos_* ≤ 2
*β_icu_*		0.1 ≤ *β_icu_* ≤ 2
*σ* ^c^	Infection rate	0.19
*υ_com_* ^d^	Probabilities of progressing to *I_com_*, *I_hos_* or *I_icu_*	0.1 ≤ *υ_com_* < 1
*υ_hos_* ^d^		0.1 ≤ *υ_hos_* < 0.25
*υ_icu_* ^d^		0.1 ≤ *υ_icu_ < υ_hos_*
*γ* ^e^	Removing rate	0.08
*μ_com_*	Probabilities of dying among *I_com_*, *I_hos_* or *I_icu_*	0 ≤ *μ_com_ < μ_hos_*
*μ_hos_*		*μ_com_ < μ_hos_ < μ_icu_*
*μ_icu_*		*μ_hos_ < μ_icu_* ≤ 1

^a^ Assumed to be 5 days (i.e., the incubation period according to Li et al. [11]) before the first case was announced in Sicily on 25 February. ^b^ Approximated to the current Sicilian population. ^c^ Assumed to be 1/5.2 days according to Li et al. [11]. ^d^ Their sum *(υ_com_* + *υ_hos_* + *υ_icu_)* was assumed to be equal to 1. ^e^ *γ* Assumed to be 1/12 days based on values reported by Wang et al., Chen et al. and Grasselli et al. [6,12,13].

**Table 2:** Best-fitting parameters in absence of control measures.

SEIRD Parameters	Definition	Estimated Values (95%CI)
*β_com_*	Transmission rate	0.99 (0.96–1.04)
*β_hos_*		0.37 (0.32–0.43)
*β_icu_*		0.28 (0.24–0.33)
*υ_com_*	Probability of progression to *I_com_*, *I_hos_*, or *I_icu_*	0.76 (0.70–0.82)
*υ_hos_*		0.19 (0.15–0.23)
*υ_icu_*		0.05 (0.03–0.07)
*μ_com_*	Probability of dying	0.01 (0.00–0.02)
*μ_hos_*		0.07 (0.04–0.10)
*μ_icu_*		0.26 (0.20–0.30)

**Table 3:** Transmission rates after the adoption of control measures.

Control Measures	Transmission Rates	Estimated Values (95%CI)
First set of restrictions adopted on $$$10 March 2020	*β_com_*	0.67 (0.57–0.76)
	*β_hos_*	0.34 (0.33–0.35)
	*β_icu_*	0.25 (0.23–0.27)
Second set of restrictions adopted on 23 March 2020	*β_com_*	0.20 (0.11–0.30)
	*β_hos_*	0.28 (0.25–0.31)
	*β_icu_*	0.22 (0.20–0.25)

## Supplementary Materials

The following are available online at https://www.mdpi.com/1660-4601/17/14/4964/s1, Figure S1: summary of data collection, model formulation and fitting, Table S1: extract of data on SARS-CoV-2 cases in Sicily (Italy), reported by the Italy’s Civil Protection from 24 February to 13 April 2020, Table S2: best-fitting parameters in the sensitivity analysis.
