# Supplementary material for: Modeling the Novel Coronavirus (SARS-CoV-2) Outbreak in Sicily, Italy
Source: Int J Environ Res Public Health. 2020 Jul 9;17(14):4964. doi: 10.3390/ijerph17144964 (PMC7400186; doi:10.3390/ijerph17144964)
Supplement: Supplementary file 1 [file ijerph-17-04964-s001.pdf]

Supplementary Materials

Modeling the Novel Coronavirus (SARS-CoV-2) outbreak in Sicily, Italy

Andrea Maugeri [1], Martina Barchitta [1], Sebastiano Battiato [2] and Antonella Agodi [1,3,*]

[1] Department of Medical and Surgical Sciences and Advanced Technologies "GF Ingrassia", University of Catania, 95123, Catania, Italy
[2] Department of Mathematics and Computer Science, University of Catania, 95123, Catania, Italy
[3] Azienda Ospedaliero-Universitaria "Policlinico-Vittorio Emanuele", 95123 Catania, Italy
* Correspondence: agodia@unict.it

Index

- **Figure S1.** Summary of data collection, model formulation and fitting
- **Table S1.** Extract of data of SARS-CoV-2 cases in Sicily (Italy), reported by the Italy's Civil Protection from 24 February to 13 April, 2020

Figure S1. Summary of data collection, model formulation and fitting

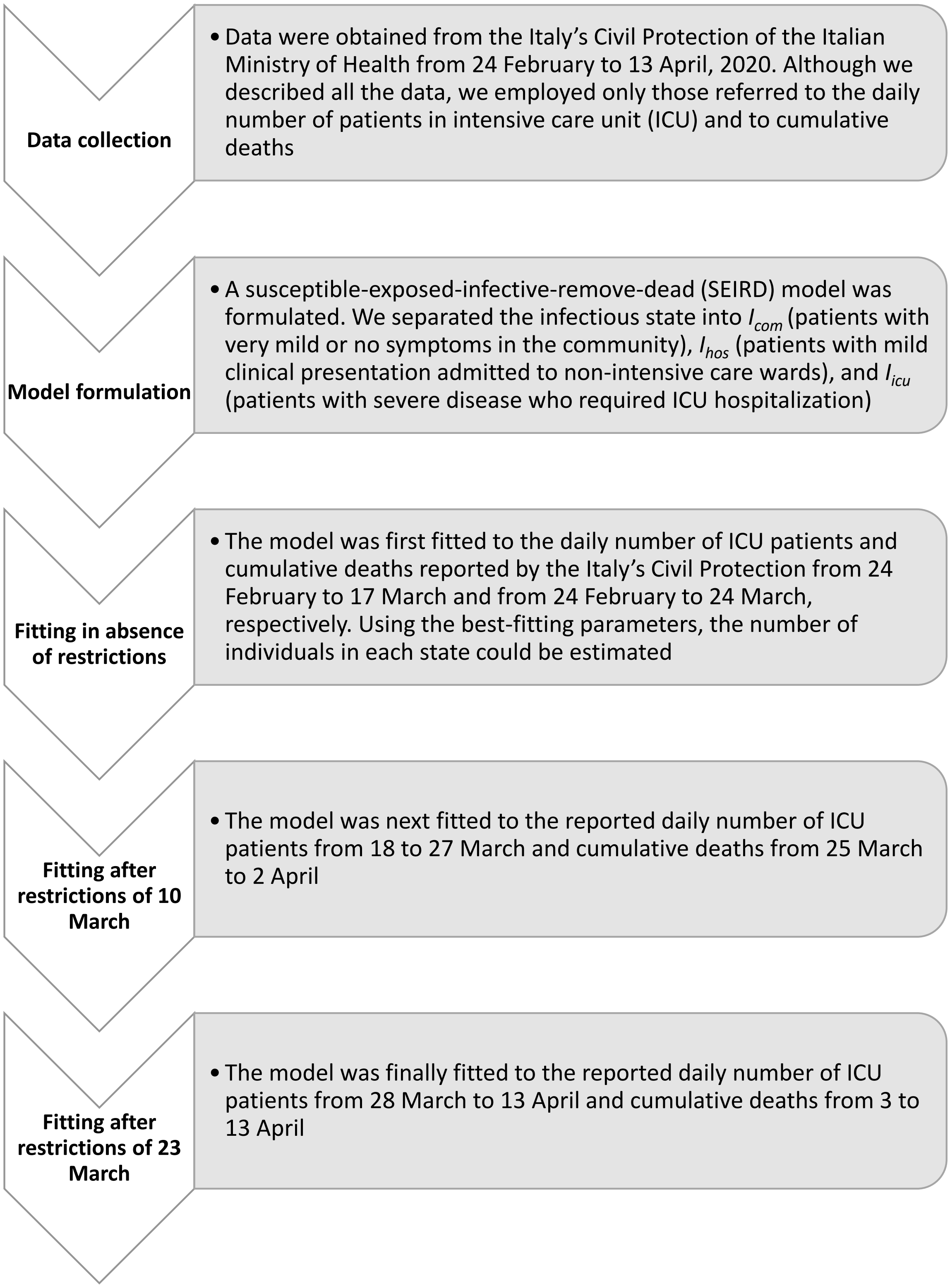

Table S1. Extract of data on SARS-CoV-2 cases in Sicily (Italy), reported by the Italy's Civil Protection from 24 February to 13 April, 2020

Date	Hospitalized in non-intensive care wards[a]	Hospitalized in Intensive Care Unit[a,b]	Total hospitalized patients[a]	Non-hospitalized patients[a]	Recovered[a]	Deaths[a,b]	Total cases[a]
**24-Feb-20**	0	0	0	0	0	0	0
**25-Feb-20**	1	0	1	2	0	0	3
**26-Feb-20**	1	0	1	2	0	0	3
**27-Feb-20**	1	0	1	1	2	0	4
**28-Feb-20**	1	0	1	1	2	0	4
**29-Feb-20**	1	0	1	1	2	0	4
**1-Mar-20**	1	0	1	6	2	0	9
**2-Mar-20**	2	0	2	3	2	0	7
**3-Mar-20**	2	0	2	3	2	0	7
**4-Mar-20**	5	0	5	11	2	0	18
**5-Mar-20**	5	0	5	11	2	0	18
**6-Mar-20**	7	0	7	15	2	0	24
**7-Mar-20**	8	0	8	25	2	0	35
**8-Mar-20**	18	0	18	33	2	0	53
**9-Mar-20**	19	0	19	33	2	0	54
**10-Mar-20**	17	2	19	41	2	0	62
**11-Mar-20**	23	1	24	57	2	0	83
**12-Mar-20**	28	5	33	78	2	2	115
**13-Mar-20**	37	7	44	82	2	2	130
**14-Mar-20**	42	11	53	97	4	2	156
**15-Mar-20**	56	15	71	108	7	2	188
**16-Mar-20**	75	20	95	108	8	2	213
**17-Mar-20**	86	28	114	112	8	3	237
**18-Mar-20**	100	29	129	138	12	3	282
**19-Mar-20**	143	36	179	142	15	4	340
**20-Mar-20**	168	42	210	169	25	4	408
**21-Mar-20**	206	48	254	204	26	6	490
**22-Mar-20**	220	55	275	321	26	8	630
**23-Mar-20**	250	60	310	371	27	13	721
**24-Mar-20**	250	60	310	489	27	20	846
**25-Mar-20**	259	80	339	597	33	25	994
**26-Mar-20**	346	68	414	681	36	33	1164
**27-Mar-20**	425	75	500	658	53	39	1250
**28-Mar-20**	441	71	512	730	60	57	1359
**29-Mar-20**	451	71	522	808	65	65	1460
**30-Mar-20**	484	75	559	849	71	76	1555
**31-Mar-20**	503	72	575	917	74	81	1647

**1-Apr-20**	496	72	568	976	86	88	1718
**2-Apr-20**	503	73	576	1030	92	93	1791
**3-Apr-20**	535	73	608	1056	94	101	1859
**4-Apr-20**	553	74	627	1099	95	111	1932
**5-Apr-20**	556	76	632	1142	104	116	1994
**6-Apr-20**	563	74	637	1178	108	123	2046
**7-Apr-20**	562	73	635	1224	113	125	2097
**8-Apr-20**	563	65	628	1265	133	133	2159
**9-Apr-20**	566	63	629	1313	152	138	2232
**10-Apr-20**	568	62	630	1337	187	148	2302
**11-Apr-20**	562	58	620	1381	209	154	2364
**12-Apr-20**	552	53	605	1425	223	163	2416
**13-Apr-20**	554	51	605	1445	237	171	2458

[a] These data are described in Figure 2 reported in the main text

[b] These data are used to fit the Susceptible-Exposed-Infectious-Recovered-Dead (SEIRD) model

Table S2. Best-fitting parameters in the sensitivity analysis.

Modeling period	SEIRD Parameters	Estimated values (95%CI)
In absence of control measures	β_{com}	0.99 (0.96 – 1.04)
	β_{hos}	0.37 (0.32 – 0.43)
	β_{icu}	0.28 (0.24 – 0.33)
	υ_{com}	0.76 (0.70– 0.82)
	υ_{hos}	0.19 (0.15 – 0.23)
	υ_{icu}	0.05 (0.03 – 0.07)
	μ_{com}	0.01 (0.00 – 0.02)
	μ_{hos}	0.07 (0.04 – 0.10)
	μ_{icu}	0.26 (0.20 – 0.30)
After restrictions adopted on 10 March, 2020	β_{com}	0.67 (0.57 – 0.76)
	β_{hos}	0.34 (0.33 – 0.35)
	β_{icu}	0.25 (0.23 – 0.27)
After restrictions adopted on 23 March, 2020	β_{com}	0.20 (0.11– 0.30)
	β_{hos}	0.28 (0.25 – 0.31)
	β_{icu}	0.22 (0.20 – 0.25)
